# 

**Published:** 1897-11

**Authors:** 


					﻿CONTENTS.
ORIGINAL ARTICLES :
Remarks of Prof. J. McF. Gaston upon Opening the Course in
Surgery at the Southern Medical College............. 531
Recurrent Appendicitis. By William C. Dugan, M. D., Louis-
ville, Ky.........................................   586
A Distinguished Physician-Pharmacist—His Great Discovery,
Ether-Anesthesia. By Joseph Jacobs................ 537
SELECTIONS AND ABSTRACTS :
A Contribution to the Therapeutics of Iron......... 546
The Treatment of Caries of the Spine............... 552
Poultices in Pulmonary Diseases of Children........ 558
A Mahar Quarter... :............................... 559
Wound of the Heart—Recovery ............................. 560
The Uses and Limitations of Condensed Milkas an Infant Food 561
The Identification of the Dead........................... 562
Round Shoulders in Girls................................. 564
Sudden Death............................................  565
Yellow Fever in the Olden Time...........................	566
Treatment of Carcinoma of the Uterus and Ovarian Disease With-
out Operation.......................................... 568
EDITORIAL:
Swear Off................................................ 571
Football................................................. 571
The Tobacco Habit Among the Young ....................... 572
Book Reviews......................................... 574,	575
				

